# Cervical Myelopathy Caused by Posttraumatic Osteophytes Resulting From Long-Standing Neglected Posterior Atlanto-Occipital Dislocation More Than 30 years: A Case Report

**DOI:** 10.5435/JAAOSGlobal-D-21-00129

**Published:** 2021-10-04

**Authors:** Dong-Gune Chang, Jong-Beom Park, Soo-Bin Park, Hong Jin Kim

**Affiliations:** From the Department of Orthopaedic Surgery, Sanggye Paik Hospital, College of Medicine, Inje University, Seoul, Korea (Dr. Chang and Dr. Kim), and the Department of Orthopaedic Surgery, College of Medicine, The Catholic University of Korea, Seoul, Korea (Dr. J.-B. Park and S.-B. Park).

## Abstract

Traumatic atlanto-occipital dislocation (AOD) is a rare but usually fatal injury. To our knowledge, no study has reported long-standing neglected posterior AOD more than 30 years in a patient who survived and later experienced cervical myelopathy.

**Methods:**

A 75-year-old man presented with symptoms of cervical myelopathy. On history, the patient was diagnosed with posterior AOD that occurred after a fall 31 years ago, but he did not undergo surgery. Radiologic evaluation of cervical spine revealed severe spinal cord compression caused by posttraumatic osteophytes of the C0-C1-C2 joints resulting from long-standing neglected posterior AOD. However, no instability of the C0-C1-C2 joints was found.

**Results:**

Laminectomy of the C1 posterior arch was performed without occipitocervical fusion considering the long-standing severe osteoarthritic changes and no instability of the C0-C1-C2 joints. Cervical myelopathy significantly improved, and the patient was doing well without recurrence at the 7-year follow-up.

**Discussion:**

To our knowledge, this is the first report of a patient with cervical myelopathy caused by neglected posterior AOD with posttraumatic osteophytes of the C0-C1-C2 joints. Laminectomy of the C1 posterior arch without occipitocervical fusion achieved satisfactory outcomes for cervical myelopathy caused by posttraumatic osteophytes resulting from long-standing neglected posterior AOD more than 30 years.

Traumatic atlanto-occipital dislocation (AOD) is one of the fatal injury but a rare condition.^1,2^ Therefore, traumatic AOD is often reported in the form of a case report.^3,4,5,6,7^ Diagnosis of traumatic AOD was usually neglected because of low clinical suspicion, severe polytrauma, and difficult to radiographic evaluation of the craniovertebral junction.^8-10^

To date, there are few reports of cervical myelopathy caused by C2 dens fracture or related complications, including nonunion, malunion, and C1-C2 instability.^11,12,13,14,15^ In addition, few reports described cervical myelopathy resulting from degenerative or hypertrophic osteoarthritis of the C1-C2 joint.^16,17,18,19,20,21,22^ To our knowledge, however, no study has reported cervical myelopathy caused by long-standing neglected traumatic posterior AOD and its late sequel involving the C0-C1-C2 joints. Therefore, in this report, we present a patient with cervical myelopathy caused by long-standing neglected posterior AOD and posttraumatic osteophytes of the C0-C1-C2 joints, which was successfully treated by laminectomy of the C1 posterior arch alone without occipitocervical fusion (OCF).

## Case Report

A 75-year-old man presented with neck pain (neck visual analog scale score: 4), bilateral radiating arm pain (arm visual analog scale score: 5/6), and gait disturbance for 3 months (Supplemental Digital Content 1, http://links.lww.com/JG9/A165). On admission, neurologic examination revealed a spastic gait, hand clumsiness, and exaggerated deep tendon reflexes in the bilateral upper and lower extremities. Pathologic Babinski sign and ankle clonus were present. Muscle strength of both upper and lower extremities was decreased. Grip and release test was performed 12 times for 20 seconds each. The modified Japanese Orthopedic Association score was 8. However, he reported no dysuria or constipation. An evaluation of the patient's trauma history revealed that he experienced severe trauma to the neck after a fall from 5 m, 31 years ago, but he experienced no neurologic deficit and any sign of spinal cord injury. He had been admitted to a local private hospital for 1 week and underwent radiographic examination. Posterior AOD was suspected at axial CT scans then, but he was discharged without receiving any surgery from the hospital because his pain improved after brace and pain medications. Since that time, he has been working as a farmer for 30 years without any significant problems but almost impossible daily activity at the time of admission.

**Figure 1:**
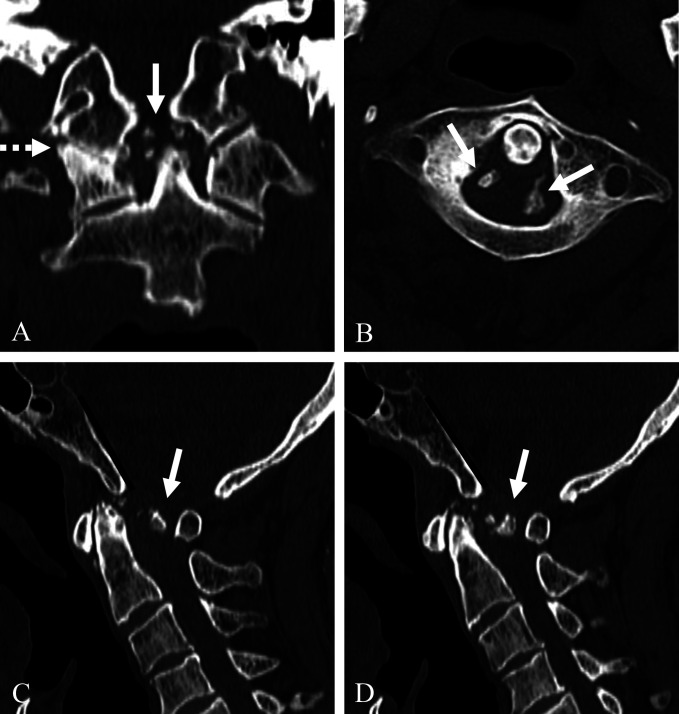
Coronal reconstructed CT (**A**) scan showing severe osteoarthritic changes (dotted white arrow) and posttraumatic osteophytes (white arrow) of the C0-1 joint. Axial CT (**B**) scan showing posttraumatic osteophytes of the C1-C2 joint (white arrows). Sagittal reconstructed CT (**C** and **D**) scans demonstrating the Wackenheim line (dark lines) behind the dens and posttraumatic osteophytes of the C0-C1-C2 joints (white arrows).

**Figure 2:**
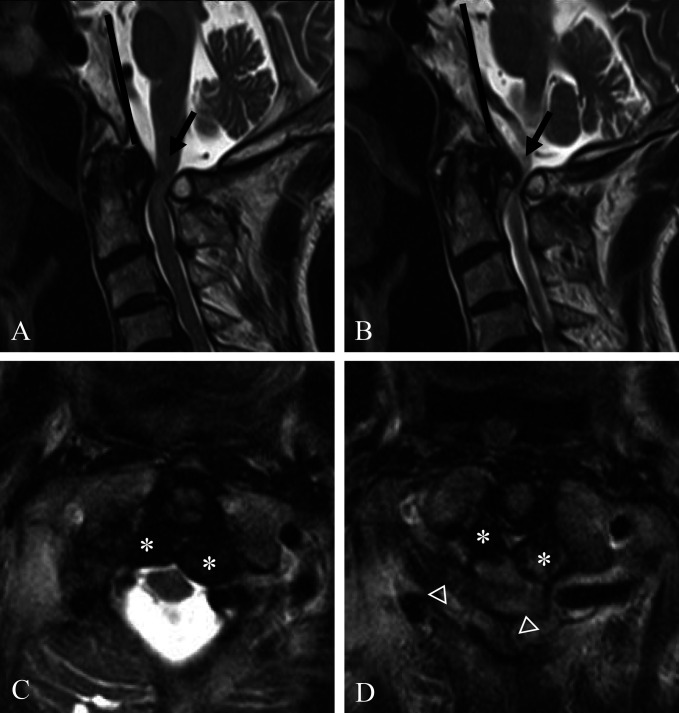
Sagittal MRI (**A** and **B**) showing the Wackenheim line (dark lines) behind the dens and severe spinal cord compression by both posttraumatic osteophytes (white arrows) and C1 posterior arch of the C0-C1-C2 joints. Axial MRI (**C** and **D**) showing severe spinal cord compression by both posttraumatic osteophytes (asterisks) and the C1 posterior arch (arrowheads).

**Figure 3:**
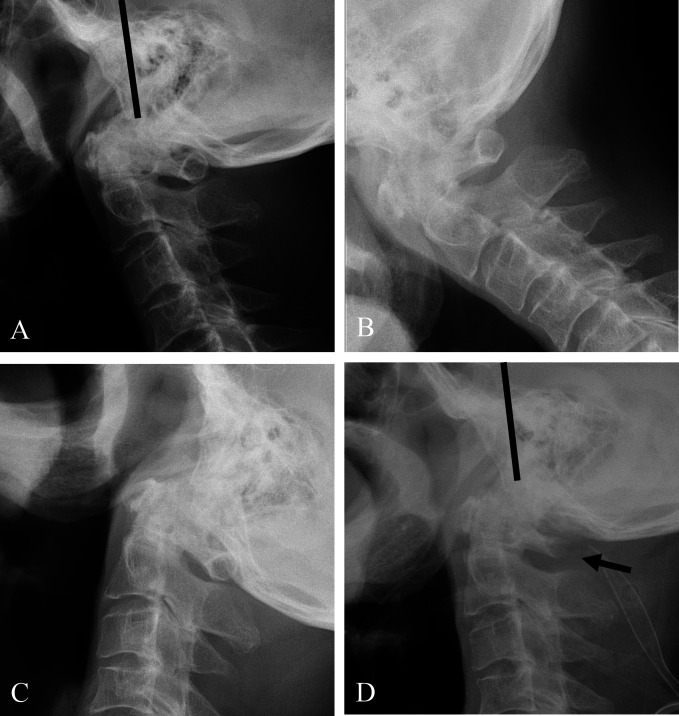
Preoperative lateral radiograph (**A**–**C**) of the cervical spine showing running of Wackenheim line (dark line) behind the dens. Postoperative lateral radiograph (**D**) of the cervical spine demonstrating laminectomy of the C1 posterior arch (dark arrow).

Coronal reconstructed CT (Figure 1, A) scan showed severe osteoarthritic change (dotted white arrow) and osteophytes (white arrow) of the C0-C1 joint. Axial CT (Figure 1, B) scan showed osteophytes of the C1-C2 joint (white arrows). Sagittal reconstructed CT (Figure 1, C and D) scans showed the Wackenheim line (dark lines) behind the dens, which indicated traumatic posterior AOD, and osteophytes of the C0-C1-C2 joints (white arrows). Sagittal MRI (Figure 2, A and B) showed the Wackenheim line (dark lines) behind the dens and severe spinal cord compression by both osteophytes and the C1 posterior arch of the C0-C1-C2 joints (white arrows). Axial MRI revealed severe spinal cord compression by both osteophytes (asterisks) and fibrous tissues and the C1 posterior arch (arrowheads) (Figure 2, C and D). Preoperative plain radiographs of the cervical spine showed the Wackenheim line (dark line) behind the dens (Figure 3, A) without significant instability of the C0-C1-C2 joints in flexion and extension (Figure 3, B and C).

**Figure 4:**
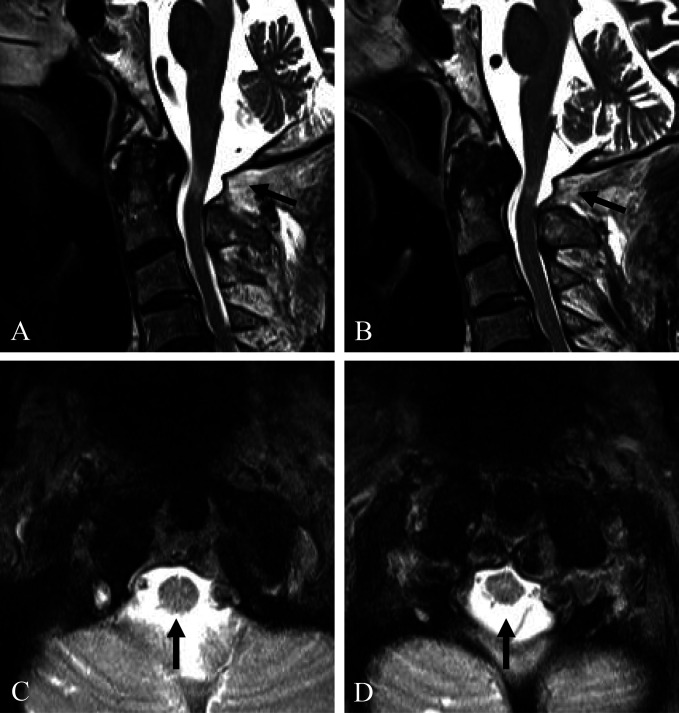
Postoperative MRI (**A**–**D**) showing decompression of the spinal cord after laminectomy of the C1 posterior arch (dark arrows).

We believe that the patient's cervical myelopathy was caused by a neglected posterior AOD with posttraumatic osteophytes of the C0-C1-C2 joints and that the C0-C1-C2 joints were stable because of long-standing severe osteoarthritic changes. Therefore, despite neglected posterior AOD, laminectomy of the C1 posterior arch alone was performed without OCF, considering patient's old age (Figure 3, D). Postoperatively, the cervical myelopathy significantly improved to modified Japanese Orthopedic Association score of 14 with a recovery rate of 66.7% (Supplemental Digital Content http://links.lww.com/JG9/A165. Postoperative MRI (Figure 4, A–d) showed decompression of the spinal cord after laminectomy of the C1 posterior arch (dark arrows). At the 7-year follow-up after surgery, the patient was doing well without recurring symptoms.

## Discussion

An accurate diagnosis of AOD is very difficult with only plain radiograph because of the unique anatomy and bony overlap of the craniovertebral junction. Reconstructed CT and MRI are essential for diagnosis of AOD in most patients.^3,8-10^ Nevertheless, an accurate diagnosis of AOD is not always established during the initial evaluation. In our patient, traumatic AOD was not definitely diagnosed only with axial CT, which was performed 30 years ago. The patient in this report experienced severe neck trauma 30 years ago that was likely the cause of posterior AOD, but no definite diagnosis was made then. We speculate that the diagnosis was overlooked because the patient experienced isolated posterior AOD without associated upper cervical spine injuries.

The cause of cervical myelopathy is very complex and multifactorial. The most underlying cause of cervical myelopathy in our patient was neglected posttraumatic AOD for 30 years, which caused the progression of posttraumatic arthritic change at C0-C1-C2 joints by secondary osteophytes and fibrous tissue, resulting in the compression of C0-C1-C2 joints. Reconstructed CT at presentation showed posterior AOD and severe osteoarthritic changes with osteophytes of the C0-C1-C2 joints. MRI demonstrated severe spinal cord compression by both osteophytes and fibrous tissue and C1 posterior arch of the C0-C1-C2 joints. If the calcified tissues (what we call posttraumatic osteophytes) detected by CT scan was acute fracture fragment caused by posttraumatic AOD, the patient may show any sign of spinal cord injury at the time of injury. However, the patient reported only neck pain without sign of spinal cord injury at the initial injury. Therefore, both calcified tissues could be considered as posttraumatic osteophyte caused by neglected posttraumatic AOD for 30 years. Therefore, taking into account the trauma history and the findings from the spinal imaging studies, we believe that the patient's cervical myelopathy was caused by posttraumatic osteophytes of the C0-C1-C2 joints resulting from neglected posterior AOD. In patients with C2 dens fracture and traumatic dislocation/subluxation, a few case reports of cervical myelopathy due to complications, including hypertrophic nonunion, malunion, and instability. However, to our knowledge, this is the first report of cervical myelopathy caused by neglected posterior AOD with posttraumatic osteophytes of the C0-C1-C2 joints in a patient who has survived for 20 years.

Traumatic AOD is typically caused by high-energy trauma. Furthermore, the report on survival in traumatic AOD is extremely rare and only showed in the form of a case report.^3,4,5,6,7^ Early surgical stabilization including OCF is recommended as the standard treatment. In the case of intractable neck pain by severe osteoarthritis of atlanto-occipital or antlato-axial joint, the neck pain could be significantly improved by OCF or atlanto-axial fusion. However, in the patient presented, we performed laminectomy of the C1 posterior arch without OCF for several reasons. First, no significant instability in the C0-C1 joint was noted on preoperative dynamic radiographs. This finding suggested that prolonged AOD, neglected for 20 years, had caused severe osteoarthritic changes of the C0-C1 joint, resulting in a stable situation. Second, the patient has lived well for 20 years as a farmer without significant problems but needed surgical intervention because daily activity was impossible after abrupt onset of cervical myelopathy. Third, the main cause of cervical myelopathy was severe spinal cord compression between posttraumatic osteophytes and C1 posterior arch. Therefore, we believed that removal of the C1 posterior arch was sufficient to achieve decompression of the spinal cord. As expected, the cervical myelopathy significantly improved after surgery, and the patient was doing well without recurring symptoms at a long-term follow-up. In conclusion, we reported the first patient with cervical myelopathy caused by neglected posterior AOD with posttraumatic osteophytes of the C0-C1-C2 joints, which was successfully treated by laminectomy of the C1 posterior arch without OCF.
